# Determining the relative contribution of retinal disparity and blur cues to ocular accommodation in Down syndrome

**DOI:** 10.1038/srep39860

**Published:** 2017-01-10

**Authors:** Lesley Doyle, Kathryn J. Saunders, Julie-Anne Little

**Affiliations:** 1 Biomedical Sciences Research Institute, University of Ulster, Cromore Road, Coleraine, Northern Ireland, BT52 1SA.

## Abstract

Individuals with Down syndrome (DS) often exhibit hypoaccommodation alongside accurate vergence. This study investigates the sensitivity of the two systems to retinal disparity and blur cues, establishing the relationship between the two in terms of accommodative-convergence to accommodation (AC/A) and convergence-accommodation to convergence (CA/C) ratios. An objective photorefraction system measured accommodation and vergence under binocular conditions and when retinal disparity and blur cues were removed. Participants were aged 6–16 years (DS n = 41, controls n = 76). Measures were obtained from 65.9% of participants with DS and 100% of controls. Accommodative and vergence responses were reduced with the removal of one or both cues in controls (p < 0.007). For participants with DS, removal of blur was less detrimental to accommodative responses than removal of disparity; accommodative responses being significantly better when all cues were available or when blur was removed in comparison to when proximity was the only available cue. AC/A ratios were larger and CA/C ratios smaller in participants with DS (p < 0.00001). This study demonstrates that retinal disparity is the main driver to both systems in DS and illustrates the diminished influence of retinal blur. High AC/A and low CA/C ratios in combination with disparity-driven responses suggest prioritisation of vergence over accurate accommodation.

In a naturalistic environment the accommodative and vergence systems use three distinct cues; retinal blur, retinal disparity and proximity, in order to achieve clear and single vision. It has been demonstrated that retinal disparity is the primary cue to both the accommodative and vergence systems in children and adults, followed by retinal blur, with proximity having little influence^1^,^2^. Additionally, accommodation is driven by vergence (and vice versa) with the neural cross-link between the two systems such that accommodation occurs as a result of convergence (convergence-accommodation) and convergence occurs as a result of accommodation (accommodative-convergence)^3^. Clinically, the relationship between the accommodation and vergence systems is often expressed using accommodative-convergence to accommodation (AC/A) and convergence-accommodation to convergence (CA/C) ratios.

An accommodative deficit is a common clinical finding in both adults and children with Down syndrome (DS)^4^,^5^,^6^,^7^,^8^,^9^,^10^,^11^, often requiring clinical intervention such as the provision of bifocal spectacle correction to minimise near visual impairment. The aetiology of hypoaccommodation in DS is not yet clear, with a variety of suggestions including: a sensory deficit^7^,^11^, a mechanical limitation^7^,^8^,^9^,^11^ or an atypical relationship between the accommodative and vergence systems^6^,^8^,^9^.

To date, no reports have evaluated the contribution of individual cues in driving the accommodative and vergence systems in DS nor the relationship between the two. The authors have previously reported simultaneous measurement of accommodation, vergence and the near pupillary response using photorefraction under naturalistic conditions, demonstrating that underaccommodation accompanies accurate vergence in the majority of participants with DS^12^. The aim of the present study is to determine the relative contribution of both the retinal disparity and retinal blur cues in driving the accommodative and vergence systems in DS and to assess the relationship between the two systems in terms of AC/A and CA/C ratio.

## Methods

### Particinpants

All experimental procedures including participant recruitment and data collection were carried out in accordance with the Declaration of Helsinki. Recruitment of 76 typically developing control participants and 41 participants with DS aged 6–16 years was carried out as described in a recently published study^12^. The study received ethical approval from the University of Ulster Research Ethics committee and the Office for Research Ethics Committees Northern Ireland, and research governance was obtained through the University of Ulster and the Research Office of the Western Health and Social Care Trust. Informed consent was obtained from the parent/guardian of all participants prior to inclusion. A clinical history, refractive error, best-corrected visual acuity (BCVA) and ocular posture status were available for all participants.

### Assessment of the Accommodative and Vergence Systems Using Photorefraction

The PowerRefractor III (PR III) (Plusoptix, Germany) eccentric photorefraction system was used to simultaneously and binocularly measure accommodative and vergence responses (50 Hz) to a range of accommodative/vergence demands and under a number of accommodative/vergence cue conditions. Refractive error ≤−0.50 DS or ≥+0.50 DS and ≤−1.00 DC were corrected either with habitual spectacles where appropriate (full distance spectacle prescription in single vision form) or using full aperture trial lenses. Where habitual spectacle correction included a bifocal segment (n = 15), full aperture trial lenses equating to the participants’ full distance spectacle prescription were used.

Refractive power and eye position calibration was attempted on all participants using spherical full aperture trial lenses and prisms of known power (+4, +3, +2, +1, −2, −4D spherical lenses and 4, 8, 12 and 16^Δ^ base in and base out prisms)^12^,^13^. Lenses/prisms were placed in front of the non-dominant eye in combination with an infrared (IR) transmitting filter (Edmund Optics, USA) to occlude visible light. This ensured participants did not perceive any change in the clarity or position of the target. Linear regression analysis of the known power of lenses/prisms against the induced change was used; the slope of which yielded a calibration value. All subsequent raw data was adjusted by each participants’ calibration value. In the event that calibration was not successful the group mean calibration was used (DS or control).

Participants viewed a commercially available animated movie target presented on a mobile LCD monitor encased within a matte black enclosure. The target stimulus contained broadband spatial frequency content in an attempt to capture and maintain attention across a wide range of ages and cognitive abilities, and to represent a naturalistic and familiar viewing experience. The target subtended 9.65° by 4.57° of visual angle when positioned 1 m from the participant before being moved to accommodative/vergence demands of 1, 2, 3, 4 and 5D/MA. The target remained stationary at each position until approximately five seconds of consistent and reliable quality data capture had been secured, during which time the participant was fixing on the target. The target was not scaled for proximity. Data capture was monitored using video recordings enabling periods of optimal cooperation to be identified for data analysis. The video files took the form of real-time video screen capture of the PR III recording with video feed interruptions used to indicate a change in target position. Video files were also used to reconfirm periods of participant attention to the target and to provide a time stamp for periods of successful and sufficient data capture, providing segments of data for further analysis.

The accommodative/vergence target was presented at demands of 1, 2, 3, 4, and 5D/MA under four cue conditions as illustrated in Table 1.

- All Cue Condition.
- Removal of the Retinal Disparity Cue (-Disparity).
- Removal of the Retinal Blur Cue (-Blur).
- Removal of both the Retinal Disparity and Retinal Blur Cues (Proximity Only).

### (i) All Cue Condition (All Cues)

Participants viewed the video target binocularly allowing naturalistic use of retinal disparity, retinal blur and proximity cues.

### (ii) Removal of the Retinal Disparity Cue (-Disparity)

A monocular protocol identical to that of the binocular protocol was carried out with the addition of an IR transmitting filter placed in front of the non-dominant or non-fixing eye. The use of the IR transmitting filter allowed occlusion of visible light to the non-dominant or non-fixing eye yet allowed continuing data collection by the PR III.

### (iii) Removal of the Retinal Blur Cue (-Blur)

In order to remove retinal blur as an accommodative/vergence cue a ‘Difference of Gaussian’ (DoG) filter was placed over the video screen^14^,^15^. The DoG filter was used to low-pass filter the stimuli edges to remove spatial frequencies greater than one cycle per degree whilst allowing colour and motion to be perceived. The audio element of the movie target also remained available and participants were encouraged to continue fixing on the video target despite its blurred appearance. The area surrounding the target screen within the enclosed surround was blocked from view of the participant in order to prevent any high spatial frequency information being available.

### (iv) Removal of Both the Retinal Disparity and Retinal Blur Cues (Proximity Only)

Both an IR transmitting filter placed in front of the participant’s non-dominant or non-fixing eye and a DoG filter over the video target were used to remove retinotopic cues (retinal disparity and retinal blur cues). Remaining cues, spatiotopic in nature, included ‘looming’ as the target moved towards the participant. For simplicity, this cue condition is referred to as the ‘proximity only’ condition.

Data analyses were performed using Matlab (Mathworks, USA) and Stata statistical software (StataCorp, USA). In brief, raw PR III data files were processed using Matlab including the application of calibration factors, removal of any data captured during blinks, filtering to remove extraneous refractive power data outside the PR III working range of −7.00 to +5.00 DS, removal of data outside the PR III pupil size working range of 4–8 mm and removal of eye position data outside ±15°^16^,^17^. A two second vignette of data was selected for analysis to calculate mean refractive power and eye position at each demand for both the right and left eyes. From gaze position and IPD, vergence in metre angles was calculated. A mean of right and left refractive power and total vergence was plotted against the accommodative/vergence demand and linear regression analysis applied to produce an accommodative and vergence response gain value (i.e. slope of the linear regression line)^3^,^12^,^14^,^15^,^20^.

### AC/A and CA/C Ratio

AC/A and CA/C ratios were derived from the accommodative and vergence response slope values from the removal of the retinal disparity (condition ii) and the removal of the retinal blur cue (condition iii) respectively^3^,^15^,^20^. Individual AC/A ratios were calculated by dividing the vergence response slope value by the accommodative response slope value taken during the removal of the retinal disparity cue condition. Similarly, the CA/C ratio was calculated by dividing each participant’s accommodative response slope value by their vergence response slope value taken during the removal of the retinal blur cue condition.

## Results

### Success Rates

Useable data were obtained from 76 control participants (100%) and 27 participants with DS (66%) for at least one or more protocols. Poor cooperation and/or intermittent reflection of the IR light source from spectacle or trial lenses resulted in the inability to collect data or the acquisition of poor quality or insufficient data. There was no significant difference in age, spherical equivalent refractive error (SER) or visual acuity between participants with DS whose data were available for analysis and those whose data were not (p > 0.05).

### Participants

Table 2 summarises the gender, mean age, SER and BCVA of participants in both the control and DS groups. All participants were Caucasian. Three participants with DS had a manifest deviation during testing, despite full refractive correction. For two participants with manifest angles less than 15^∆^ (one alternating exotropia and one esotropia), accommodative responses were averaged from the right and left eyes^3^. In the case of one participant where a larger angle was present, all data from the non-fixing eye were removed during the filtering process as a result of eye position criterion used (removal of eye position data outside ±15°)^16^. As a result, it was not possible to generate a measure of vergence for this individual and accommodative data were obtained only from the fixing eye to avoid off-axis errors^3^. Participants with DS were younger than control participants (t_(97)_ = 2.68, p = 0.009), significantly more hyperopic (SER OD: t_(96)_ = −1.98, p = 0.03, SER OS: t_(96)_ = −2.27, p = 0.01) and had significantly poorer BCVA (better seeing eye: t_(96)_ = −11.22, p < 0.00001).

### Accommodative Response

**Figure 1:**
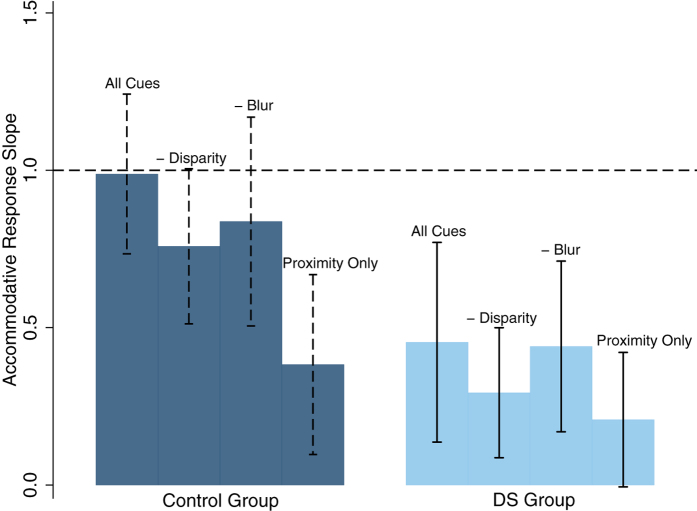
Mean accommodative response slope across each of the cue conditions for both control participants and participants with DS. Error bars are representative of the standard deviation. Control participants are represented in navy and participants with DS in light blue. The dashed line represents a perfect response slope value of 1.

Table 3 and Fig. 1 summarise the mean (±SD) accommodative response slope values under each cue condition for each participant group. Statistical analysis revealed a significant difference in accommodative response slope values between cue conditions (two-way ANOVA, F_(2,3)_ = 31.40, p < 0.00001) and between groups (F_(2,1)_ = 141.59 p < 0.00001). This model also demonstrated a significant interaction between cue conditions by participant group (F_(2,3)_ = 5.46, p = 0.001). Table 4 summarises the interactions between cue conditions for each participant group, using post-hoc analysis. Control participants had significantly more accurate accommodative response slope values in all four cue conditions in comparison to participants with DS (p < 0.01). Accommodative response slope values were significantly reduced with the removal of one (retinal disparity or retinal blur) or both cues (retinal disparity and retinal blur) in control participants. For participants with DS the removal of the blur cue was less detrimental to accommodative response values than the removal of disparity, with accommodative response slope values significantly better when all cues were available or when the blur cue was removed in comparison to when proximity was the only available cue. These findings remained unchanged when participants with DS and strabismus were removed (n = 3).

Due to participants with DS being significantly more hyperopic in comparison to control participants a refractive error matched control group (REM Controls) were also used for comparison. Each participant with DS (n = 27) was matched to one or more participants from the control group who had SER within ±1.00 DS of that of the respective participant with DS for both eyes (n = 34). Accommodative response slope values remained significantly more accurate in REM control participants in comparison to DS participants (two-way ANOVA, F_(1,1)_ = 87.83, p < 0.00001) across all cue conditions (p < 0.01) and a significant interaction between cue conditions and participant group remained (F_(2,3)_ = 3.30, p = 0.02).

### Vergence Response

**Figure 2:**
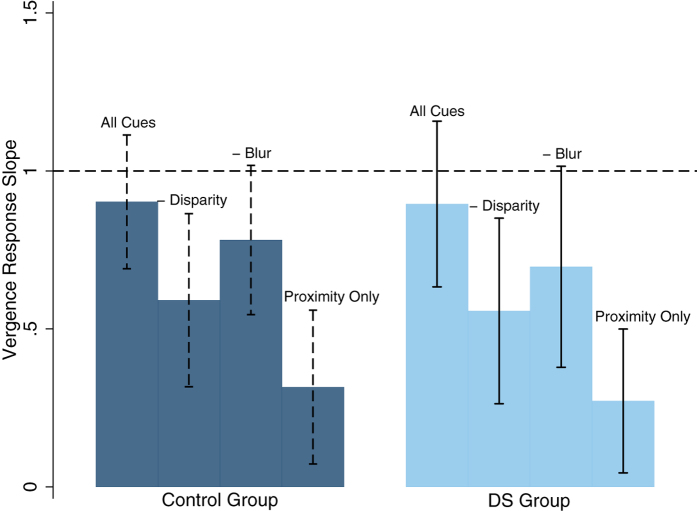
Mean vergence response slopes for each cue condition for both groups. Errors bars are representative of the standard deviation. Control participants are represented in navy and participants with DS in light blue with a dashed line representing a perfectly accurate response slope of 1.

Table 5 and Fig. 2 summarise the mean (±SD) vergence response slope values across the four cue conditions by participant group. There was a significant difference in vergence response slope value between cue conditions (two-way ANOVA, F_(2,3)_ = 70.36, p < 0.00001) but no significant difference between groups (p = 0.85). This remained unchanged when REM controls were used and when participants with DS and manifest strabismus were removed. Table 6 summarises the interactions between cue conditions for both control and DS participants. Vergence response slope values were significantly reduced in the control group with the removal of one cue (either retinal disparity or retinal blur) or when both cues (retinal disparity and retinal blur) were removed. For participants with DS vergence response slopes were also significantly reduced with the removal of retinal disparity alone or with the removal of both retinal disparity and retinal blur. The removal of the retinal blur cue alone did not significantly reduce vergence response slope values in participants with DS.

### AC/A and CA/C Ratio

**Figure 3:**
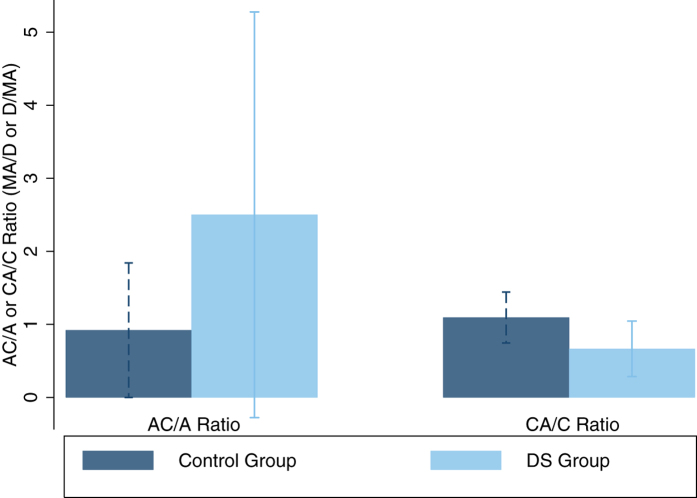
Mean AC/A and CA/C ratios by participant group. Error bars are representative of the standard deviation. Control participants are represented in navy and participants with DS in light blue.

Figure 3 illustrates AC/A and CA/C ratios by participant group. Mean AC/A ratios were 0.92 ± 0.92MA/D (range = 0.25–8.14MA/D) and 2.50 ± 2.78MA/D (range = 0.02–12.81MA/D) for the control group and participants with DS respectively. Participants with DS had significantly larger AC/A ratios in comparison to control participants (two-group mean comparison, t = −4.17, p < 0.00001), remaining unchanged when REM controls were used for comparison (t = −3.30, p = 0.0009) and when participants with DS and manifest strabismus were removed (t = −4.14, p < 0.00001). Mean CA/C ratios were 1.09 ± 0.35D/MA (range = 0.10 to 2.10D/MA) and 0.69 ± 0.39D/MA (range = 0.15–1.39D/MA) for control participants and participants with DS respectively. Participants with DS had significantly smaller CA/C ratios in comparison to control participants (two-group mean comparison, t = 4.86, p < 0.00001), remaining unchanged when REM controls were used for comparison (t = 3.10, p = 0.002) and when participants with DS and manifest strabismus were removed (t = 4.11, p < 0.00001).

## Discussion

This is the first study to simultaneously assess the accommodative and vergence responses in children with DS with the removal of one, or both of the main cues to the accommodation and vergence systems; retinal disparity and retinal blur. This investigation enables scrutiny of the relationship between the two systems in terms of AC/A and CA/C ratios. The results demonstrate that retinal blur contributes little to the accommodative and vergence responses exhibited by participants with DS. The data also illustrate that participants with DS demonstrate significantly larger AC/A and smaller CA/C ratios in comparison to their typically developing peers.

A number of previous studies have used a variety of experimental procedures in order to remove and isolate accommodative and vergence cues and to ascertain their individual contribution to the accommodative and vergence responses of typically developing individuals^1^,^2^,^15^,^18^,^19^,^20^. When investigating the use of cues in adults (both naïve and expert observers) and children using experimental procedures similar to those of the present study, Horwood and Riddell^2^,^15^,^21^,^22^ found that retinal disparity was the primary cue to both the accommodative and vergence systems followed by retinal blur. This finding was replicated for both typically developing control participants and those with DS in the present study. However, results from participants with DS reveal an insensitivity to retinal blur and demonstrate that retinal disparity is more influential in driving both the accommodation and vergence systems.

Horwood and Riddell^21^ suggest that individuals whose responses are driven by the retinal disparity cue may be more tolerant of imprecise accommodation. A sensory mechanism has also been suggested as explanation of the underaccommodation demonstrated in DS^7^,^11^, in that individuals with DS may have an increased depth of focus or increased tolerance of retinal blur, allowing underaccommodation to occur without the perception of an out-of-focus image. The authors previously demonstrated a significant relationship between BCVA and the accommodative response in children with DS; as BCVA worsened so did the accuracy of the accommodative response^12^. It may be hypothesised that if participants with DS have degraded visual acuity and thus a reduced ability to resolve high spatial frequency information, this may result in an insensitivity to small changes in retinal blur and consequently its lack of contribution to the accommodative and vergence systems.

The present study demonstrates that participants with DS have significantly larger AC/A ratios and significantly smaller CA/C ratios in comparison to their typically developing peers. Horwood and Riddell^3^ suggest that AC/A and CA/C ratios can further reveal how retinal disparity and retinal blur drive responses. However, two individuals can have the same AC/A or CA/C ratio but their underlying accommodation and vergence profile can be markedly different. When considering participants with DS, the high AC/A ratio is the result of low levels of accommodation and relatively normal vergence, rather than as a result of accurate accommodation and excessive convergence, as seen in some forms of convergence excess esotropia. The AC/A and CA/C ratios demonstrated in children with DS suggest strong accommodation-vergence coupling and week convergence-accommodation coupling. Cregg *et al*.^7^ previously suggested that convergence driven accommodation may be reduced or absent in children with DS. A reduction or absence of convergence driven accommodation could result in an accurate vergence response accompanied by underaccommodation, as shown in the present study. The abnormal relationship between the accommodation and vergence systems and resultant AC/A and CA/C ratios demonstrated by participants with DS may prevent these individuals from achieving retinal images that are simultaneously clear and single. The accommodative and vergence systems in DS are more responsive to retinal disparity cues, thus it appears that the DS visual system prioritises the attainment of a single image and accurate vergence over a clear retinal image and accurate accommodation. Based on the ratios demonstrated in participants with DS, if a clear retinal image were given preference, this would encourage over-convergence and potentially the development of esotropia, a common finding in children with DS^5^,^8^,^23^,^24^,^25^.

Woodhouse *et al*.^5^ demonstrated reduced accommodative responses from three months of age in children with DS, the age at which the accommodative system becomes adult-like for typically developing infants^26^. It has been suggested that the cross-coupling of the accommodative and vergence systems recalibrates over time from infancy to adulthood in order to compensate for emmetropisation and an increase in inter-pupillary distance with age^1^. However, it is known that the DS eye often fails to emmetropise^24^,^25^ and it is unclear what impact this failure may have on the development and nature of the relationship between the accommodative and vergence systems. Prospective data from infancy are required to explore whether underaccommodation and normal vergence responses supported by high AC/A and low CA/C ratios are present during early visual development in DS.

A number of studies have demonstrated that the use of bifocal correction can result in an improvement in the accuracy of accommodative responses in children with DS^26^,^27^. Their findings, in combination with the results of the present study suggest that by reducing the accommodative demand, bifocal correction may allow participants with DS to experience single and clear vision, without the accommodative and vergence systems being in conflict with one another. This sensory visual experience may facilitate recalibration of accommodative-vergence coupling. Longitudinal study of the accommodative and vergence systems in DS are required to determine whether the relationship between the two systems is abnormal from infancy and whether the cross-coupling recalibrates over time with refractive or bifocal correction.

## Conclusion

In conclusion, this is the first study to simultaneously assess the use of individual cues in driving both the accommodative and vergence responses in DS and the relationship between the two systems in terms of AC/A and CA/C ratio. The data demonstrate that retinal disparity is the main driver to both the accommodative and vergence systems in DS and illustrate the diminished influence of retinal blur cues to accommodation and vergence in DS, both indicative of a sensory deficit of the accommodative system. High AC/A and low CA/C ratios in combination with disparity-driven responses also suggest an abnormal relationship between the two systems, with accurate vergence prioritised at the expense of accurate accommodation.

## Additional Information

**How to cite this article**: Doyle, L. *et al*. Determining the relative contribution of retinal disparity and blur cues to ocular accommodation in Down syndrome. *Sci. Rep.* **7**, 39860; doi: 10.1038/srep39860 (2017).

**Publisher's note:** Springer Nature remains neutral with regard to jurisdictional claims in published maps and institutional affiliations.

## Figures and Tables

**Table 1:** Table illustrating the availability of accommodative and vergence cues in each cue condition.

Cue Condition	Availability of Cues		
	Retinal Disparity	Retinal Blur	Proximity
(i) All Cues	Yes	Yes	Yes
(ii) Disparity	No	Yes	Yes
(iii) Blur	Yes	No	Yes
(iv) Proximity Only	No	No	Yes

**Table 2:** Table summarising the gender, mean age (±SD), mean (±SD) and range of refractive error and VA of the best and worst seeing eye in participants with and without DS.

Group	Gender	Mean Age (Years)		Mean SER (D)	SER Range (D)	BCVA (logMAR)
DS (n = 27)	9 males	9.88 ± 2.95	Better Seeing Eye	1.99 ± 2.45	−2.25 to +7.63	0.25 ± 0.16
			Worse Seeing Eye	2.14 ± 2.56	−2.00 to +7.88	0.34 ± 0.19
Controls (n = 76)	36 males	11.51 ± 3.17	Better Seeing Eye	0.72 ± 2.23	−6.00 to +8.25	−0.10 ± 0.08
			Worse Seeing Eye	0.75 ± 2.14	−5.25 to +7.25	−0.03 ± 0.12

**Table 3:** The mean (±SD) and range of accommodative response slope values for each cue condition in each participant group.

Group	Protocol	No. of Participants	Mean Accommodative Response Slope Value	±SD	Range of Accommodative Response Slope Values
DS	(i) All Cues	25 (62.0%)	0.46	0.31	0.04–1.19
	(ii) Removal of Disparity	23 (56.1%)	0.29	0.21	−0.02–0.83
	(iii) Removal of Blur	22 (53.7%)	0.44	0.27	0.03–0.84
	(iv) Proximity Only	22 (53.7%)	0.21	0.21	−0.13–0.80
Controls	(i) All Cues	75 (98.7%)	0.99	0.25	0.45–1.70
	(ii) Removal of Disparity	76 (100%)	0.76	0.25	0.07–1.67
	(iii) Removal of Blur	73 (96.1%)	0.84	0.33	−0.20–1.73
	(iv) Proximity Only	74 (97.4%)	0.38	0.29	−0.07–1.21

**Table 4:** Table summarising the interactions of accommodative response slopes between cue conditions by participant group.

Group	Cue Condition Interactions			
	Cue Condition	p-value		
		All Cues	- Disparity	-Blur
DS	- Disparity	0.19		
	- Blur	1.00	0.33	
	Proximity Only	**0.01**	1.00	**0.02**
Controls	- Disparity	**<0.0001**		
	- Blur	**0.007**	0.53	
	Proximity Only	**<0.0001**	**<0.0001**	**<0.0001**

Statistically significant interactions are highlighted in bold print.

**Table 5:** Table summarising the mean (±SD) vergence response slope value for each cue condition and each participant group.

Group	Protocol	No. of Participants	Mean Vergence Response Slope Value	±SD	Range Vergence Response Slope Values
Controls	(i) All Cues	75 (98.7%)	0.90	0.21	0.47–1.44
	(ii) Removal of Disparity	75 (98.7%)	0.59	0.27	−0.44–1.54
	(iii) Removal of Blur	72 (94.7%)	0.78	0.24	−0.12–1.33
	(iv) Proximity Only	72 (94.7%)	0.32	0.24	−0.18–0.93
DS	(i) All Cues	22 (53.7%)	0.90	0.26	0.51–1.62
	(ii) Removal of Disparity	21 (51.2%)	0.56	0.29	0.006–1.03
	(iii) Removal of Blur	20 (48.8%)	0.70	0.32	−0.03–1.22
	(iv) Proximity Only	21 (51.2%)	0.27	0.23	−0.05–0.82

**Table 6:** Table summarising the vergence response slope interactions between cue conditions for both groups using two-way ANOVA.

Group	Cue Condition Interactions			
	Cue Condition	P-Value		
		All Cues	- Disparity	-Blur
DS	- Disparity	**0.001**		
	- Blur	0.14	0.66	
	Proximity Only	**<0.0001**	**0.008**	**<0.0001**
Controls	- Disparity	**<0.0001**		
	- Blur	**0.02**	**<0.0001**	
	Proximity Only	**<0.0001**	**<0.0001**	**<0.0001**

Statistically significant interactions are highlighted in bold print.
